# Pattern of Presentation and Utilization of Services for Mental and Neurological Disorders in Northeastern Nigeria: A Ten-Year Study

**DOI:** 10.1155/2015/328432

**Published:** 2015-11-24

**Authors:** Jidda Mohammed Said, Abdulmalik Jibril, Rabbebe Isah, Omeiza Beida

**Affiliations:** ^1^Department of Psychiatry, College of Medicine, University of Maiduguri, PMB 1069, Nigeria; ^2^Department of Psychiatry, College of Medicine, University of Ibadan, PMB 5116, Nigeria; ^3^Federal Neuropsychiatry Hospital Maiduguri, PMB 1322, Nigeria; ^4^Federal Neuropsychiatry Hospital Kaduna, Badarawa, Kaduna, Nigeria

## Abstract

Mental and neurological disorders are common in the primary health care settings. The organization of mental health services focuses on a vertical approach. The northeast as other low income regions has weak mental health services with potentially huge mental health burden. The manner of presentations and utilization of these services by the population may assist in determining treatment gap. We investigated the pattern and geographical distribution of presentations with mental disorders and explored the linkages with primary care in northeastern Nigeria over the last decade. A retrospective review of hospital-based records of all the available mental health service units in the region was conducted over a decade spanning between January 2001 and December 2011. A total of 47, 664 patients attended available mental health facilities within the past decade in the northeast. Overwhelming majority (83%, *n* = 39,800) attended the region's tertiary mental health facility. A substantial proportion (30%, *n* = 14,440) had primary physical illness, while 18%, *n* = 8606, had primary neurologic disorders. The commonest physical comorbidity was hypertension (4%) and diabetes (2%). A significant proportion of the populace with mental disorders appeared not to be accessing mental health care services, even when it is available. Meaningful efforts to improve access to mental health services in the northeast region of Nigeria will require successful integration of mental health into primary and general medical services.

## 1. Introduction

Mental, neurological, and substance use (MNS) disorders account for an estimated 14% of the global burden of disease [1, 2]. These disorders result in direct economic costs of mental health care and indirect economic costs from lost productivity, impaired functioning, and premature death [3].

Evidence suggests that the burden of mental and neurological disorders predominates in the primary health care settings [4]. Mental disorders are known to appear with different somatic presentations in primary care settings with features of neurological disorders and contribute significantly to the hidden burden of mental diseases [5]. 25% of primary care patients have unrecognized mental health disorders. It is estimated elsewhere to be between 13 and 51% using instruments of varying criteria of diagnosis [6, 7]. Emphasis is being placed on horizontal rather than vertical approach to service delivery [8]. Scaling of mental and neurological health care services which is also advocated especially in middle and low income countries to address the needs in primary care settings requires adequate specialist manpower [9].

However, across the majority of low and middle income countries, a low level of skilled mental health professionals is the norm [4–7]. This is particularly true for highly skilled mental health service providers such as psychiatrists and psychologists [2, 8]. This situation is also true for Nigeria [9] with significant intracountry disparity in the distribution of mental health personnel resources [10]. The northeastern region of Nigeria is the least resourced, with a weak mental health system that is poorly funded and has very few mental health professionals, as compared to the rest of the country. This intracountry disparity and relatively poor funding are buttressed by the fact that only 1% of the regional health budget is allocated to mental health compared to the 3% national average [9, 11]. Furthermore, the region's 0.069 psychiatrists per 100,000 population are a far cry from the 0.15/100,000 national average.

Available mental health care services are limited to a single tertiary mental health facility and psychiatric units in 5 general hospital settings in the entire northeastern region of Nigeria, comprising 6 states and serving a population of nearly 20 million people. Additionally, these mental health facilities also provide services to substantial parts of the neighbouring countries of Chad, Cameroon, and Niger republics.

The life time prevalence of mental illness among Nigerians is 12.1% and the 12-month prevalence is 5.2%; and while 20% of these are severe enough to warrant hospital admission, only 8% of serious and debilitating mental illnesses are actually treated [9]. In absolute terms, this means that, for the northeast region with a population of about 20 million, some 2.42 million people will suffer one form of mental illness or another in their life time and 208,000 individuals will require hospitalization in the northeast every year. This is potentially a huge burden upon the limited available mental health resources in the northeast.

While the resources for mental health care services are clearly limited in northeast Nigeria, the burden of mental disorders is high and is comparable to what is observed in most low and middle countries [12]. Paradoxically, underutilization of available services due to multiple layers of barriers is often the norm in much of middle and low income countries [7, 13, 14] (Figure 4).

Although the weakness of the mental health system of the region is evident and the needs for mental and neurological care services are high it is not known to what extent the available services are currently being utilized. It is also not known to what extent the needs for services remain unmet; the extent of communal utilization of the available mental health services is not known. This information is useful to provide useful insights and a basis for future regional mental health policy formulation, planning, or the implementation of national policies at the local regional level. It will also assist in achieving a nuanced understanding of the mental health gap of the region.

This study therefore aimed at investigating the pattern and geographical distribution of presentation with mental disorders, along with an exploration of the linkages with primary care in northeastern Nigeria over the last decade.

## 2. Methods

A retrospective review of hospital-based records of all the available mental health service units in the region was conducted over a decade spanning between January 2001 and December 2011.

*Study Setting.* We studied the mental health services of the northeastern region of Nigeria comprising 6 states of Adamawa, Bauchi, Borno, Gombe, Taraba, and Yobe (Table 2). The centres where data were collected were the Federal Neuropsychiatry Hospital, Maiduguri, Borno state, and the psychiatric units of the specialist hospitals located in the state capitals of the states. The Federal psychiatric Hospital is a tertiary mental health facility which has a computerized health information department. The psychiatric units maintain a mental case register of diagnosis, treatment, and biodata of patients. Two health records staffs were recruited to extract the data from the hospital database in Maiduguri, Borno state. In the psychiatric units psychiatric nurses were trained on how to extract the data from the mental case registers into the proforma.

*Sample.* The hospital records of the total population of patients attending the 5 mental health units of general hospitals and the 1 specialist mental health hospital in the northeast from January 2001 to December 2011 were studied.

*Inclusion Criteria.* All patients whose records had adequate information that included biodata, diagnosis, and treatment offered were recruited into the study.

*Procedure*. A proforma was designed to capture the data required for the study which included the biodata, diagnosis, treatment, year of first presentation, and the mental health facility where treatment was received. Research assistants were recruited to fill in the information which was then transferred onto SPSS for statistical analysis. Only those facilities with the required information were included. One of the psychiatry units in a general hospital had very poor records and was therefore excluded. However, a cursory evaluation revealed that the numbers from the excluded hospital did not significantly differ from the others.

Ethical clearance was obtained from the Ethics and Research Committee of the tertiary facility while permission was obtained from the relevant management of all the health facilities utilized in this study. This was a health system research that did not require identifying information such as names of the individual clients. Thus, we could guarantee that the anonymity of the individual patients was preserved.

## 3. Results

The sociodemographics of the population of patients utilizing the mental health services in the northeast in the period under review in Table 1 showed more females (54%) accessing the services. Most were unemployed (73.3%) and 43% were illiterate. A total of 47,664 patients were attended to in all mental health facilities within the past decade in the northeast. An overwhelming majority (83%, *n* = 39,800) were handled by the region's tertiary mental health facility. A substantial proportion (30%, *n* = 14,440) had primary physical illness, while 18%, *n* = 8606, had primary neurologic disorders. The commonest physical comorbidity was hypertension (4%) and diabetes (2%).

**Figure 1:**
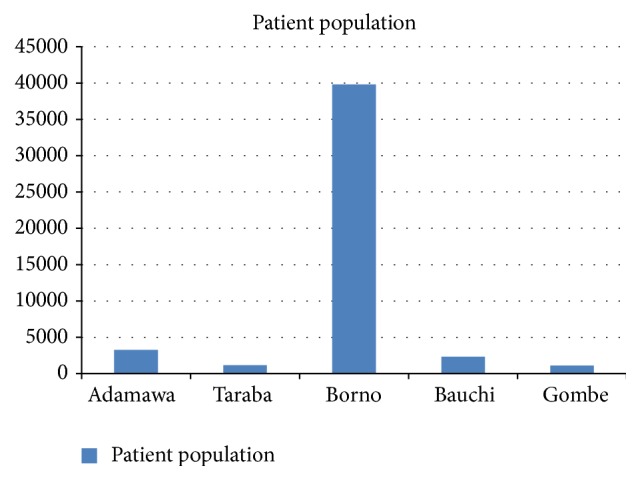
Patient populations across the region over the last decade.

The overwhelming proportions of the patients were managed in Maiduguri, which is the solitary tertiary mental health facility in the region. The specialist hospital in Taraba saw the least number of patients over the last decade. See Figure 1.

**Figure 2:**
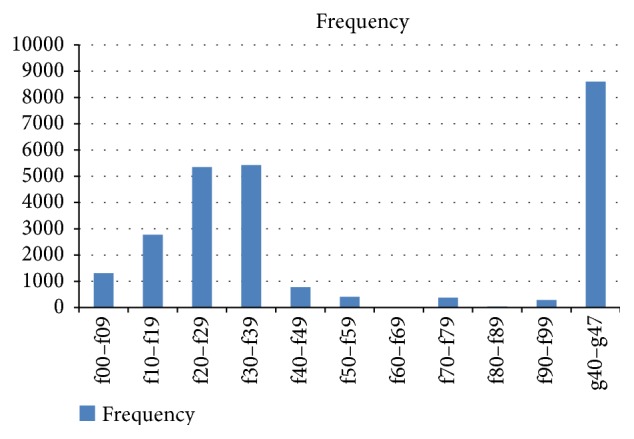
Showing distribution of patient by diagnosis. ICD-10: Classification of Mental Disorders, f00–9: Organic Mental Disorders, f10–19: Substance Abuse, f20–29: Schizophrenia Spectrum and Delusional Disorders, f30–39: Mood Disorders, f40–49: Neurotic Stress Related and Somatic Disorders, f50–59: Behavioural Syndromes associated with physiological or physical disturbance, f60–69: Personality Disorders, f70–79: Mental Retardation, f80–89: Disorders of Psychological Development, f90–98: Behavioural and Emotional Disorders with Childhood Onset, and f99: Unspecified Mental Disorders.

Primary neurological disorders were the most frequently seen conditions, followed by schizophrenia spectrum and depressive disorders, respectively. No personality disorders were diagnosed within this period. See Figure 2.

Most of the disorders were handled by the tertiary mental health facility. Relatively few were seen at the mental health facilities in the general hospitals even though they are closer to the community.

Primary physical health problems had a disproportionate representation in the out-patient clinic attendance, with 30% of the patients attending the out-patient clinics of the mental health facilities presenting with primarily physical health problems.

*Linkage of Mental Health with the Primary Health Care System.* The linkage with the regional primary health care system was poor. Only one of the secondary mental health facilities had regular linkage networks and a referral system, with the tertiary mental health centre located in Maiduguri. There was no interaction or linkages between the secondary care and the primary care level.

## 4. Discussions

The total number of patients that utilized the facilities in the ten-year period falls short of the potential number of people in need of admission for a year only. This may be due to different factors at play preventing people from accessing the services being provided. These factors include lack of awareness [7, 15], stigma, [16] and cultural factors [13]. Cultural factors play a role in symptom presentation as well as awareness of the possibility of cure for experiences which may be considered beyond the capacity of orthodox medical practice [17, 18]. The pathway to care is known to be rather tortuous with only few of the severely ill making it to the orthodox mental health centres [19, 20]. Furthermore, previous studies from three different regions of Nigeria had established that most of the mentally ill persons are more likely to try alternative treatment options before eventually accessing mental health services if things do not improve [21, 22].

**Figure 3:**
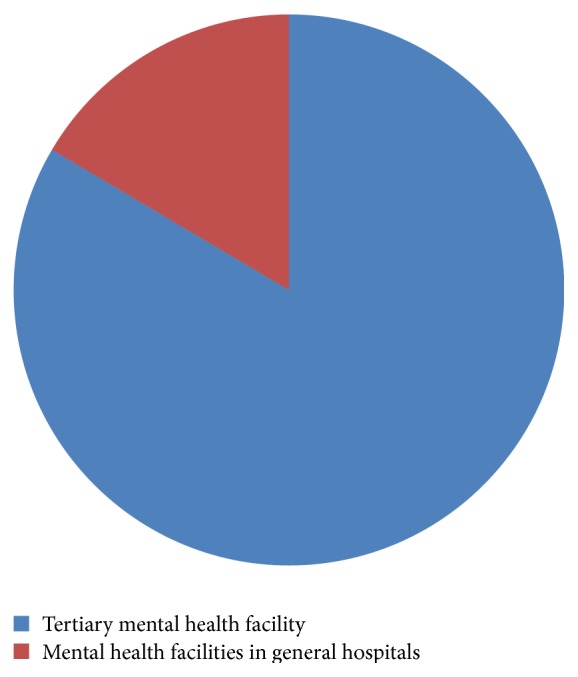
Relative utilization of health facilities over the last decade.

**Figure 4:**
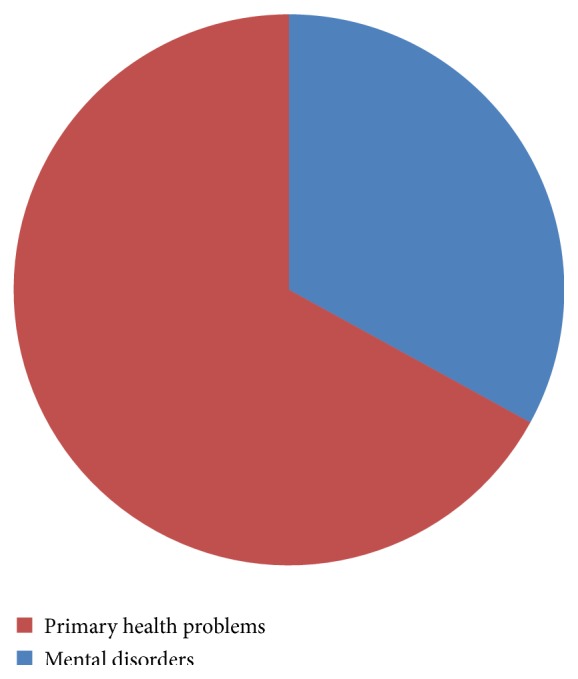
Proportion of mentally ill patients attending the mental health facilities in the region.

Although psychosis constitutes low prevalence within the community [9], schizophrenia alone appears to be the most diagnosed mental disorder reflecting not just the perception of the public concerning what constituted mental health but also the level of expertise of the service providers who may fail to diagnose cases that were nonpsychotic in presentation (Figure 3). Personality disorders, which require a certain degree of psychological awareness amongst the population, an adequate number of qualified clinicians, and the availability of other trained personnel such as clinical psychologists to ascertain the diagnosis, were reported in only 3 instances over the entire decade under review. This was particularly the case in the mental health units in the secondary care level where medical officers with interest or limited training in mental health are the frontline managers of mental health cases. This may partly explain the relatively low patronage these centres receive from the community. Conversely, it may also appear that less severe mental disorders may improve with the interventions from the spiritual/traditional healers and thus may not be presenting at the mental health facilities, while the more severely disturbed psychotic cases end up getting to hospitals because they are unlikely to improve without appropriate medications.

Our study reveals the relatively higher burden on the tertiary health facility even though it is located in the regional capital which is geographically far from the majority of the region's population and a lower rate of utilization of the psychiatric units within general hospitals, which should be closer to the populace. This may be related to the poorly skilled manpower in these facilities or a lack of awareness that such services are available in the general hospitals.

Headaches and the epilepsies (g40–g47) were seen in unusually large numbers. This may have been due to a massive public education campaign about the availability of services for this group of disorder through the electronic media over the last decade particularly the Federal Neuropsychiatry Hospital in Maiduguri, Borno state.

It is also evident that very little has changed in terms of the policy recommendation of strengthening and integrating mental health into primary care in Nigeria, even though the country's mental health policy was passed over two decades ago (1991) as evidenced by the low levels of utilization of the psychiatric units located in the psychiatric units located outside the regional capital. It will appear that the pragmatic way forward to increase access to mental health care services in underserved populations such as the northeastern region of Nigeria will have to be based on the fulcrum of effective integration of mental health care services into primary and general medical care services. This will however require adequate training in mental health, as well as ongoing supportive supervision from the few available mental health professionals from the tertiary facility.

## 5. Conclusion

Overall there is poor utilization of in-patient services by the population majority of whom were treated at the tertiary health centre. A significant proportion of the populace with mental disorders appeared not to be accessing mental health care services, even when it is available. Meaningful efforts to improve access to mental health services in the northeast region of Nigeria will require successful integration of mental health into primary and general medical services. Community awareness and mobilization to engage with and to utilize these services where available will also be pertinent.

## Figures and Tables

**Table 1:** Sociodemographic characteristics of the patients utilizing mental and neurologic services in northeast between 2001 and 2010.

	Sample *n*	Percentage (%)
Sex		
Male	21925	46%
Female	25739	54%
Marital status		
Married	28646	60.1%
Single	16349	34.3%
Divorced	1954	4.1%
Widowed	715	1.5%
Education		
Primary	19065	40.0%
Secondary	5052	10.6%
Tertiary	2860	6.0%
None	21449	43.4%
Occupation		
Employed	12726	26.7%
Not employed	34938	73.3%

**Table 2:** Utilization of mental and neurological services in the northeastern Nigeria between 2001 and 2011.

	In-patient	Out-patient
	*n* (%)	*n* (%)
Centres		
Adamawa	207 (15.0)	2299 (85.0)
Borno	3951 (9.1)	35821 (90.9)
Bauchi	306 (17.6)	2054 (82.4)
Gombe	161 (9.7)	1166 (90.3)
Taraba	211 (11)	1488 (89)
Total	4836	42828
